# The Nobel Prize honours the discovery of the natural barrier against autoimmunity – a breakthrough in tolerance reconstitution: how far are we from clinical impact?

**DOI:** 10.1016/j.ero.2026.02.013

**Published:** 2026-03-05

**Authors:** Jens Y. Humrich, Gabriela Riemekasten

**Affiliations:** Department of Rheumatology and Clinical Immunology, University Medical Center Schleswig-Holstein/University of Lübeck, Lübeck, Germany

## INTRODUCTION

Since the early days of immunology, scientists have dreamed of treating autoimmune and immune-mediated diseases by rebalancing the immune system back to a physiological state. Thirty years after their initial discovery, it has now become a doctrine that regulatory T cells (Tregs) are indispensable constituents for the maintenance of peripheral self-tolerance and immune homeostasis by controlling and suppressing autoimmunity and harmful immune responses. Characterised by the expression of the lineage-specific transcriptional repressor forkhead-box-protein P3 (FoxP3), Tregs keep the organism in a self-tolerant and balanced state despite the presence of autoreactive immune cells. Tregs are a heterogeneous cell subset with diverse functions that include their capability to regulate the activation, differentiation, and effector functions of various immune cells and to promote the resolution of tissue inflammation and tissue repair. The discovery of this natural barrier against autoimmunity, therefore, represents one of the most intriguing breakthroughs in the history of immunology and medicine. This achievement was particularly extraordinary considering that it occurred at a time when the concept of cell-mediated immunosuppression was largely buried by most immunologists due to limited convincing evidence and methodological restrictions in defining ‘suppressor’ cells.

Unimpressed by these reservations and driven by an enormous passion, curiosity, and perseverance, Shimon Sakaguchi continued to strive for exploring cell-mediated mechanisms of peripheral immune tolerance and how their failure contributed to the development of autoimmunity. His tremendous efforts were fruitful and led to the discovery of a CD4^+^ T cell subset with a potent capacity to prevent organ-specific and systemic autoimmunity that was phenotypically characterised by the constitutive expression of the interleukin-2 (IL-2) receptor alpha-chain CD25 [1]. To distance his discovery from the discredited former concept of ‘suppressor cells’ and to prospectively acknowledge their functional diversity in regulating immune homeostasis and autoimmunity, this unique cell type was named ‘regulatory T cell’. Six years later, his fundamental discovery was complemented and confirmed at a molecular level by Mary E Brunkow and Fred Ramsdell, who demonstrated that mutations in the *FOXP3* gene, also known as Scurfin, caused the lethal poly-autoimmune syndrome observed in so-called scurfy mice and the corresponding immune dysregulation polyendocrinopathy enteropathy X-linked (IPEX) poly-autoimmune syndrome in humans [2,3]. Subsequent research based on these discoveries finally approved the critical role of the *FOXP3* gene for Treg development and function, establishing FoxP3 as the lineage-defining marker for naturally occurring and thymic-derived Treg [4,5].

As a result of these seminal findings, the new concept of immunoregulation was born, which turned out to stay on the stage, evidenced by the explosive amount of publications in this field, reaching more than 60,000 up to now. These pioneering works sparked an unbowed enthusiasm for a new research frontier aiming at harnessing Treg-based immunotherapies to reconstitute peripheral tolerance in autoimmune diseases and to prevent organ transplant rejections. Research over the past decades focusing on the role of Treg in disease pathogenesis revealed indeed that many autoimmune and rheumatic diseases involve disruptions in Treg biology, either in their numbers or their functionality, which ultimately leads to an imbalance between Treg and pathogenic immune cells. Although definite clinical approval is still pending, remarkable translational and clinical efforts are currently ongoing to make the dream come true of correcting dysregulated immune responses in a smart fashion without the necessity of harshly suppressing the immune system. Treg-based immunotherapies primarily aim to correct this imbalance by increasing the quantity or the quality of Treg in the patient. This can be achieved either through adoptive transfers of *ex vivo* expanded autologous or genetically engineered Treg or by using molecules that are capable of regenerating and expanding the Treg population directly in the patient’s body. Regarding its function in the immune system, the cytokine IL-2 was shown to play a vital and nonredundant role in the biology of Treg because it is essentially required for the growth and survival of Treg in the peripheral lymphatic tissues and for their development in the thymus. A large proportion of the Treg population constitutively expresses the trimeric, high-affinity IL-2 receptor, which renders them more sensitive to IL-2 exposure than other cell types. Therefore, the use of low doses of either native or mutated IL-2 is considered an attractive and feasible treatment modality to selectively stimulate and expand the Treg population *in vivo*. Driven by 2 independent landmark studies simultaneously published in 2011 [6,7], the therapeutic use of low doses of the ‘regulatory’ cytokine IL-2 has since emerged as the most advanced strategy within the field of Treg-based immunotherapies. This is not only highlighted by the remarkable number of clinical studies, including larger phase 2 randomised controlled clinical trials (RCTs) that have been conducted to date across more than 30 autoimmune and inflammatory diseases [8]. In addition, in some autoimmune diseases, such as systemic lupus erythematosus (SLE), a deficiency of IL-2 develops during disease pathogenesis, which leads to a disturbance of Treg homeostasis and loss of the highly suppressive Treg subset expressing CD25 at high levels (CD25^hi^ Treg). In this context, it could be demonstrated that low-dose IL-2 therapy very efficiently corrects this acquired Treg dysfunction and restores the balance between Treg and pathogenic immune cells [[8], [9], [10]]. The discovery of the mechanism underlying such Treg ‘exhaustion’ in autoimmune diseases and its reversibility provided a pathophysiologically driven rationale for the implementation of low-dose IL-2 therapy as a targeted treatment modality for autoimmune diseases, particularly in SLE (Fig.). Indeed, the level of evidence for the clinical efficacy of low-dose IL-2 therapy is most advanced and robust in SLE [8], underlined by findings from early pilot studies initiated more than 12 years ago [[10], [11], [12], [13], [14]] and from 3 more recent phase 2 RCTs that consistently demonstrated clinical benefit in SLE [[15], [16], [17]]. Interestingly, and somewhat unexpectedly from a classical immunologist’s perspective, the fatal and poorly treatable neurodegenerative disease amyotrophic lateral sclerosis (ALS) was effectively modulated by low-dose IL-2 therapy, as demonstrated in a large randomised phase 2b trial [18]. On the basis of these highly promising results, both the European Medicines Agency (EMA) and the US Food and Drug Administration (FDA) have granted this treatment an orphan drug designation (ODD) for ALS. It is therefore plausible that ALS could become the first condition for which low-dose IL-2 therapy receives regulatory approval in the near future.

**Figure:**
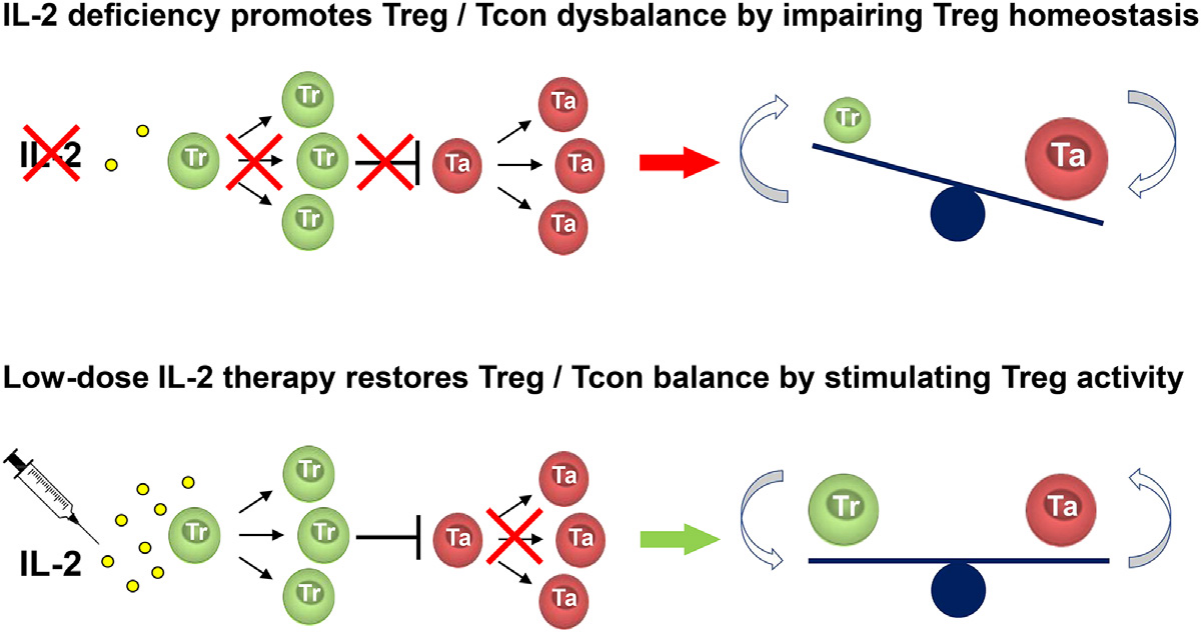
The Treg-IL-2 axis in the pathophysiology and treatment of autoimmune diseases. In autoimmune diseases, such as SLE, repression of IL-2 production due to chronic activation of Tcon leads to an acquired deficiency of the Treg growth and survival factor IL-2, which impairs the homeostasis of Treg and their suppressive capacity. This Treg insufficiency facilitates the uncontrolled expansion and chronic activation of autoreactive Tcon and promotes a dysbalance between Treg and autoreactive Tcon (upper panel). Compensation of IL-2 deficiency by low-dose IL-2 therapy stimulates expansion of the Treg population and recovers their suppressive capacity, thereby restoring the balance between Treg and autoreactive Tcon (lower panel). IL-2, interleukin-2; SLE, systemic lupus erythematosus; Ta, autoreactive T cell; Tcon, conventional T cell; Tr/Treg, regulatory T cell.

Ongoing basic and translational research will continue to illuminate the diverse functions of Treg. These efforts are expected to enhance our understanding of their mode and timing of action in the body and to further substantiate and exploit their therapeutic potential, paving the way for the clinical introduction of Treg-based immunotherapies in the future.

The reward of the Nobel Prize in Physiology and Medicine 2025 for the exceptional and game-changing discoveries on Treg, representing also a unique example of applied immunology, was somehow overdue and is more than well-deserved. This honour is in particular due to Shimon Sakaguchi, who is recognised by all experts in the field as the godfather of the Treg and the iconic founder of a new, vibrant era of immunoregulation and tolerance research. Box 1 and Box 2

Box 1 **Regulatory T cells (Tregs): from discovery to clinical application**

 

• For decades, immunologists aspired to treat autoimmune disease by restoring immune balance rather than unselectively suppressing the immune system. This vision became tangible with the discovery of Tregs, a specialised T cell subset essential for maintaining peripheral self-tolerance and controlling autoimmunity.
• In 1995, Shimon Sakaguchi identified CD4⁺CD25⁺ T cells capable of preventing systemic and organ-specific autoimmunity at a time when cell-mediated immunosuppression was widely dismissed. To distinguish them from the abandoned concept of “suppressor cells”, these cells were named regulatory T cells. Molecular validation followed when mutations in the *FOXP3* gene were shown to cause lethal autoimmunity in scurfy mice and the human immune dysregulation polyendocrinopathy enteropathy X-linked (IPEX) syndrome, establishing forkhead-box-protein P3 (FoxP3) as the lineage-defining transcription factor of Treg.
• Subsequent work revealed interleukin-2 (IL-2) as an essential and nonredundant factor for Treg survival and function. Since the publication of 2 seminal pilot studies in 2011, low-dose IL-2 therapy has advanced into multiple clinical trials, with particularly strong evidence in systemic lupus erythematosus and amyotrophic lateral sclerosis. These discoveries exemplify the successful translation of immune tolerance approaches from fundamental biology to clinical application.

Alt-text: Unlabelled box dummy alt text

Box 2 **Timeline**

 

• 1995 – CD4⁺CD25⁺ regulatory T cell (Treg) discovered
• 2001-2003 – The *FOXP3* gene is identified as a Treg lineage-defining transcription factor.
• 2011 – First pilot trials published evaluating low-dose interleukin 2 (IL-2) therapy in immune-mediated diseases.
• Present – Accumulating evidence for the clinical efficacy and very favourable safety profile of low-dose IL-2 therapy from multiple clinical trials in diverse indications, including larger randomised controlled trials (RCTs); orphan drug designation (ODD) for amyotrophic lateral sclerosis (ALS).

Alt-text: Unlabelled box dummy alt text

## CRediT authorship contribution statement

**Jens Y. Humrich:** Writing – review & editing, Writing – original draft, Visualization, Conceptualization. **Gabriela Riemekasten:** Writing – review & editing, Writing – original draft, Conceptualization.

## Competing interests

JYH reports a relationship with the German Research Foundation that includes funding grants. Gabriela Riemekasten reports a relationship with the German Research Foundation that includes funding grants. JYH reports a relationship with the European Commission that includes funding grants. JYH reports a relationship with MSD Sharp & Dohme GmbH that includes consulting or advisory, funding grants, and speaking and lecture fees. JYH reports a relationship with Sanofi-Aventis Deutschland GmbH that includes: funding grants and speaking and lecture fees. JYH reports a relationship with GentiBio that includes: board membership and consulting or advisory. JYH reports a relationship with ILTOO Pharma that includes: nonfinancial support. GR reports no conflict of interests.
